# SWATH-MS dataset of heat-shock treated *Drosophila melanogaster* embryos

**DOI:** 10.1016/j.dib.2016.11.028

**Published:** 2016-11-16

**Authors:** Bertrand Fabre, Dagmara Korona, Daniel J.H. Nightingale, Steven Russell, Kathryn S. Lilley

**Affiliations:** a Cambridge Centre for Proteomics, Department of Biochemistry, University of Cambridge, Cambridge, U.K; b Department of Biochemistry, University of Cambridge, Cambridge, U.K; c Department of Genetics, University of Cambridge, Cambridge, U.K; d Cambridge Systems Biology Centre, University of Cambridge, Cambridge, U.K

**Keywords:** *Drosophila melanogaster*, Heat shock, Mass-spectrometry, SWATH

## Abstract

Data independent acquisition (DIA) has emerged as a promising mass spectrometry based approach, combining the advantages of shotgun and targeted proteomics. Here we applied a DIA approach (termed SWATH) to monitor the dynamics of the *Drosophila melanogaster* embryonic proteome upon heat-shock treatment. Embryos were incubated for 0.5, 1 or 3 h at 37 °C to induce heat-shock or maintained at 25 °C. The present dataset contains SWATH files acquired on a Sciex Triple-TOF 6600. A spectral library built in-house was used to analyse these data and led to the quantification of more than 2500 proteins at every timepoint. The files presented here are permanent digital maps and can be reanalysed to search for new questions. The data have been deposited with the ProteomeXchange Consortium with the dataset identifier PRIDE: PXD004753.

**Specifications Table**

**Table:** 

Subject area	*Biology.*
More specific subject area	*Proteomics, Drosophila melanogaster, Heat-shock.*
Type of data	*Table, figure.*
How data was acquired	*Mass spectrometry, SWATH acquisition mode on a Sciex Triple-TOF 6600.*
Data format	*Analysed.*
Experimental factors	*SWATH-MS analysis of a heat-shock time course on Drosophila melanogaster embryos.*
Experimental features	*Drosophila melanogaster embryos were subjected to heat-shock for different times, collected, lysed, digested with trypsin and analysed in SWATH acquisition mode on a Sciex Triple-TOF 6600. The data were analysed using Spectronaut*^TM^.
Data source location	*Cambridge, United Kingdom.*
Data accessibility	*SWATH data have been deposited with the ProteomeXchange Consortium via the PRIDE partner repository with the dataset identifier PRIDE:*PXD004753.

**Value of the data**

• Heat-shock time course experiment with *Drosophila melanogaster* embryos useful to study the dynamics of the heat-shock response.
• Deep proteome coverage with more than 2500 proteins identified (FDR<0.001%) and quantified at every time points with expected heat-shock proteins regulation useful for developing and testing SWATH-MS data analysis tools/workflows.
• SWATH permanent digital maps that can be searched with new hypotheses (using optimized spectral library, analysis of the post-translational modifications, etc.).

## 1 Data

**Fig. 1:**
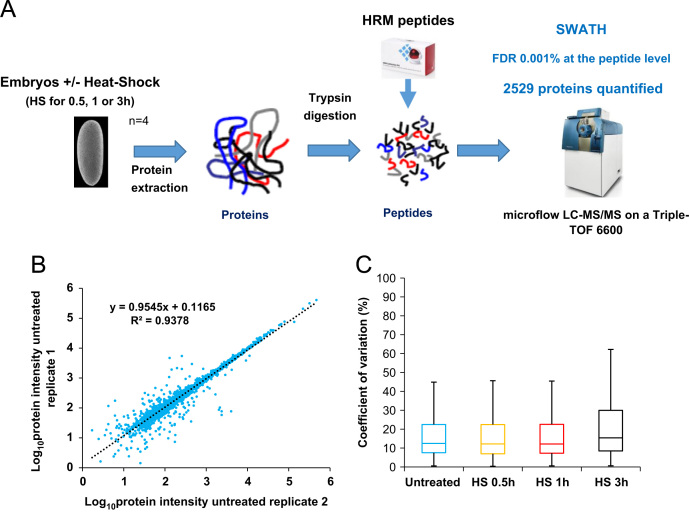
A) Overall strategy to characterize the dynamics of the *D. melanogaster* embryonic proteome after heat-shock treatment (HS) using SWATH-MS. Embryos were collected and treated for 0.5, 1 or 3 h at 37 °C or untreated. Proteins were extracted, digested with trypsin and HRM peptides were spiked into the samples before injection. The samples were analysed using SWATH acquisition mode on a Sciex Triple-TOF 6600. The resulting files were analysed with Spectronaut^TM^. 2529 proteins were quantified using this workflow. B) Reproducibility of the protein intensity measurements between biological replicates. The Log_10_ transformed protein intensities were plotted for replicates 1 and 2 of the untreated condition. C) The Coefficient of variation (CVs) between the biological replicates were calculated for each condition.

Protein expression levels of *Drosophila melanogaster* embryos were monitored by SWATH-MS [1] after heat-shock treatments. Proteins were extracted from embryos using a total lysis with a buffer containing 4% SDS. Samples were prepared using a GeLCMS preparation method and proteins were digested with trypsin. HRM peptides were spiked into the samples before analysis. The peptides were analysed using a SWATH acquisition mode on a Sciex triple-TOF 6600. The workflow used in this study is illustrated in Fig. 1A. The dataset presented here includes all the SWATH raw files and output files from the Spectronaut analysis (Supplementary table).

## 2 Experimental design, materials and methods

### 2.1 Fly lines, embryo collection and protein extraction

Adult flies of the sequenced *D. melanogaster* iso-1 strain from the Bloomington Stock Centre (*y*^*1*^*; Gr22b*^*iso-1*^*Gr22d*^*iso-1*^*cn*^*1*^*CG33964*^*iso-1*^*bw*^*1*^*sp*^*1*^*; LysC*^*iso-1*^*MstProx*^*iso-1*^*GstD5*_*iso-1*_*Rh6*^*1*^) were kept on standard yeast-cornmeal media in a 12-h light-dark cycle at 25 °C and 75% relative humidity. Embryos were collected from cages on apple juice agar plates 16 h after egg laying. After collection, embryos were incubated at 37 °C for 0.5, 1 or 3 h or retained at 25 °C for 3 h. The timepoints chosen in this study were adapted from [2]. The embryos were then dechorionated with 50% bleach, washed with water, frozen in liquid nitrogen and kept at −80 °C. Four independent biological replicates were collected for each time point. Embryos were lysed in Tris 50 mM pH 7.5, 4% SDS, protease inhibitor (Complete, Roche) using a Dounce homogenizer (50 strokes per sample). Each sample was then heated for 5 min at 95 °C. The samples were sonicated (Bioruptor (Diagenode), position high, 30 s on, 30 s off for 10 min), centrifuged at 14,000 g for 10 min and the pellets were discarded.

### 2.2 Sample preparation for mass-spectrometry analysis

In gel digestion was used for sample preparation as described in [3]. HRM peptides (Biognosys) were added to each sample before injection on the mass spectrometer.

### 2.3 Generation of the spectral library

For the generation of the SWATH assay library, a high pH reverse phase fractionation of 1 mg of a protein sample from embryos collected over a 24 h period was performed. Peptides were loaded onto an Acquity bridged ethyl hybrid C18 UPLC column (Waters, 2.1 mm inner diameter 150 mm, 1.7 µm particle size), and separated with a gradient from 0 to 35% buffer B in 50 min, and 35–100% buffer B in 7 min (buffer A: 20 mM NH4-formate pH10; buffer B: 20 mM NH4-formate pH10/80% ACN) at a flow rate of 0.244 ml min^−1^. Chromatographic performance was monitored by sampling the eluate with a diode array detector (Acquity UPLC, Waters) scanning between wavelengths from 210 to 400 nm. Fractions were collected at 1 min intervals. Thirty-two fractions were collected, dried with a Speed-Vac and resuspended in 3% acetonitrile/0.1% formic acid and analysed in Information Dependent Acquisition (IDA) mode on a TripleTOF 6600 mass spectrometer (Sciex). Half of the peptides from each fraction were injected per run. To each peptide sample, HRM peptides (Biognosys) were added before analysis. Mass spectrometry analyses were performed on a TripleTOF 6600 mass spectrometer fitted with a Duospray ion source (Sciex) and coupled to an ACQUITY UPLC System (Waters). The samples were injected onto a MicroLC column (150 mm longx0.3 mm inner diameter) with ChromXP C18CL, 300 Å pore size, 3 μm diameter particles (Sciex). Samples were run using a 49 min gradient from 3–40% solvent B (solvent A 0.1% formic acid, 5% DMSO in water; solvent B: 0.1% formic acid, 5% DMSO in acetonitrile) at a flow rate of 5 µl/min. Data were acquired using an ion spray voltage of 5.5 kV, curtain gas at 25 psi and nebulizer gas at 10 psi. A IDA method was set up with the MS survey range set between 350 and 1250 m/z (250 ms accumulation time) followed by dependent MS/MS scans with a mass range set between 100 and 2000 m/z (100 ms accumulation time) of the 20 most intense ions in the high sensitivity mode with a 2+ to 5+ charge state. Dynamic exclusion was set for a period of 15 s and a tolerance of 50 ppm. Rolling collision energy was used.

The .wiff files were analysed with MaxQuant [4] version 1.5.2.8 with the same parameters as described in [6].

The library was built using Spectronaut 8 from Biognosys [5], using the default settings, from the resulting combined file from the MaxQuant analysis. The library contains 40963 peptides corresponding to 5348 protein groups. The output files from the MaxQuant analysis have been deposited with the ProteomeXchange Consortium via the PRIDE partner repository, along with SWATH data, under the dataset identifier PXD004753.

### 2.4 SWATH data acquisition

The SWATH MS analysis was performed on a Triple-TOF 6600 as described in [6].

### 2.5 SWATH data analysis

Spectronaut 8 (Biognosys) was used to analyse the SWATH experiments. Default settings were used except for the retention time prediction type that was set to dynamic iRT with a correction factor for window of 2. A Q-value of 10^−5^ (corresponding to a FDR of 0.001% at the peptide level) was used. Proteins with at least two peptides were used for quantitative analysis. The sum of peptides intensity was used as protein intensity. Good measurement reproducibility of intensities was observed between the biological replicates as illustrated in the Fig. 1B. Using our analytical workflow, the median coefficient of variation between the biological replicates are between 12 and 15% for the different time points (Fig. 1C). Fold changes were calculated by comparing the intensities of the proteins at each time point to the untreated sample (Supplementary table).

### 2.6 Data availability

All the mass spectrometry data have been deposited with the ProteomeXchange [7] Consortium via the PRIDE partner repository with the dataset identifier PRIDE: PXD004753.

The results from the SWATH analysis of the heat-shock time course experiment are provided in Supplementary Table.
